# Early clinical experience utilizing scintillator with optical fiber (SOF) detector in clinical boron neutron capture therapy: its issues and solutions

**DOI:** 10.1186/s13014-016-0680-0

**Published:** 2016-08-09

**Authors:** Masayori Ishikawa, Tetsuya Yamamoto, Akira Matsumura, Junichi Hiratsuka, Shin-Ichi Miyatake, Itsuro Kato, Yoshinori Sakurai, Hiroaki Kumada, Shubhechha J. Shrestha, Koji Ono

**Affiliations:** 1 Department of Biomedical Science and Engineering, Graduate School of Health Science, Hokkaido University, N-12 W-5 Kita-ku, Sapporo, Hokkaido 060-0812 Japan; 2 Faculty of Medicine, University of Tsukuba, Ibaraki, 305-8575 Japan; 3 Department of Radiation Oncology, Kawasaki Medical School, Okayama, 701-0192 Japan; 4 Department of Neurosurgery, Osaka Medical College, Osaka, 569-0084 Japan; 5 Graduate School of Dentistry, Osaka University, Osaka, 565-0871 Japan; 6 Research Reactor Institute, Kyoto University, Osaka, 590-0494 Japan

**Keywords:** SOF detector, Ultra-miniature detector, Thermal neutron monitor, Clinical trial BNCT

## Abstract

**Background:**

Real-time measurement of thermal neutrons in the tumor region is essential for proper evaluation of the absorbed dose in boron neutron capture therapy (BNCT) treatment. The gold wire activation method has been routinely used to measure the neutron flux distribution in BNCT irradiation, but a real-time measurement using gold wire is not possible. To overcome this issue, the scintillator with optical fiber (SOF) detector has been developed. The purpose of this study is to demonstrate the feasibility of the SOF detector as a real-time thermal neutron monitor in clinical BNCT treatment and also to report issues in the use of SOF detectors in clinical practice and their solutions.

**Material and methods:**

Clinical measurements using the SOF detector were carried out in 16 BNCT clinical trial patients from December 2002 until end of 2006 at the Japanese Atomic Energy Agency (JAEA) and Kyoto University Research Reactor Institute (KURRI).

**Results:**

The SOF detector worked effectively as a real-time thermal neutron monitor. The neutron fluence obtained by the gold wire activation method was found to differ from that obtained by the SOF detector. The neutron fluence obtained by the SOF detector was in better agreement with the expected fluence than with gold wire activation. The estimation error for the SOF detector was small in comparison to the gold wire measurement. In addition, real-time monitoring suggested that the neutron flux distribution and intensity at the region of interest (ROI) may vary due to the reactor condition, patient motion and dislocation of the SOF detector.

**Conclusion:**

Clinical measurements using the SOF detector to measure thermal neutron flux during BNCT confirmed that SOF detectors are effective as a real-time thermal neutron monitor. To minimize the estimation error due to the displacement of the SOF probe during treatment, a loop-type SOF probe was developed.

## Background

Boron neutron capture therapy (BNCT) is the combination of external irradiation (thermal neutrons or epithermal neutrons) and internal irradiation (α particle and lithium nuclei). In other words, a boron ^10^B compound is selectively introduced into tumor cells and is externally irradiated with thermal or epithermal neutrons. The thermal neutrons interact with ^10^B in the tumor cells and result in high linear energy transfer (LET) α and lithium ^7^Li particles through boron neutron capture reaction ^10^B(n,α)^7^Li. The very short range of α particles (~8 μm) and ^7^Li particles (~5 μm) helps to destroy ^10^B loaded tumor cells at the cellular level with minimum damage to neighboring ^10^B unloaded normal cells. The first clinical trial of BNCT was carried out by Farr et al. at Brookhaven National Laboratory (BNL) in 1951 but the results were not satisfactory [1]. Later, Hatanaka et al. performed clinical trials on 13 brain tumor patients at Hitachi Training Reactor (HTR) from 1968 to 1975. The encouraging outcomes stimulated interest in BNCT [2]. In 1987, Mishima et al. started a clinical trial of BNCT for malignant melanoma using Kyoto university reactor KUR [3]. In a clinical study carried out by Nakawaga and Hatanaka on 149 patients treated with BNCT between August 1968 and April 1995 considered BNCT as an ideal treatment for malignant brain tumors because of the quality of life after treatment [4]. Currently, BNCT offers the most effective treatment for primary and metastatic tumors, specifically glioblastoma multiforme and malignant melanomas for which effective therapy has not yet been developed [5]. However, the procedure for BNCT is one of the most complex cancer treatment modalities and the effectiveness of this therapy depends on the neutron and boron distribution [6]. In clinical BNCT, the continuous monitoring of the neutron flux distribution is necessary because the distribution and intensity may vary depending on the reactor condition or the physical feature of the patients [7]. Their accurate and real-time assessment during irradiation is also essential for the quality assurance of the treatment.

Detectors that are used for thermal neutron monitoring include ^10^BF_3_ or ^3^He gas counters, ionization chambers, fission chambers and proton-recoil spectrometers [8]. The gold wire or foil activation method is also used for the same purpose. Gas-filled detectors and activation spectrometry are considered as the primary tool for neutron beam dosimetry and monitoring in BNCT. Gas filled detectors are commonly used for phantom measurement but for in-vivo measurement they are not frequently used because of their large physical size and high sensitivity to electric noise. Gold wire is the most common method used to measure thermal neutron fluence in-vivo dosimetry. Other radiation technologies have also been developed for the same purpose, such as scintillators, thermoluminescent dosimeters (TLD), gel detectors and self-powered neutron detectors [9–12].

In most BNCT clinical cases, thermal neutron fluence has been measured by means of the gold wire activation method. However, the real-time measurement of thermal neutron flux using this method is not possible since neutron activation of the gold wire alone requires at least several minutes. In our previous work, we developed a plastic scintillator with optical fiber (SOF) for online thermal neutron measurements in BNCT [9, 13]. Details of the characteristics and properties of the SOF detector can be found in our former study. Initially, we used a boron compound as a neutron converter in the scintillator and two clinical measurements were performed using a boron loaded SOF detector. The output this detector showed good agreement with gold wire measurements but the measured value comprised of much electric noise and was latter replaced by a LiF mixed scintillator.

In the present research, measurements were carried out on a total of 16 patients using the SOF detector, until the end of 2006 at Japan Atomic Energy Agency (JAEA) / JRR4 and Kyoto University Research Reactor Institute (KURRI). In the first two clinical cases boron loaded scintillators were used, and in the remaining 14 cases LiF mixed scintillators were used. The main purpose of this study was to demonstrate the feasibility of the SOF detector as a real-time thermal neutron monitor during BNCT treatment based on results of clinical measurements. We also report in this paper about issues we experienced in the use of SOF detectors in clinical BNCT practice and their solutions.

## Materials and methods

### Measurement system

**Fig. 1:**
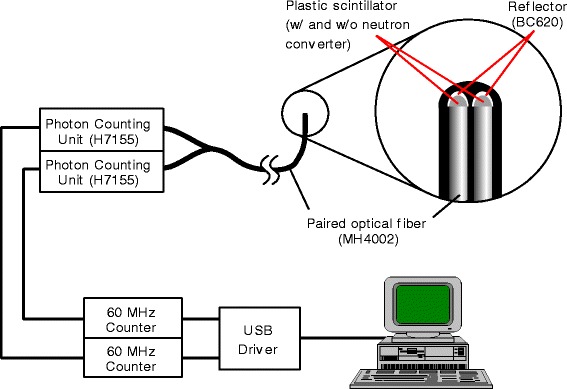
Schematic diagram of the paired SOF detector system. The signal processing of the paired SOF detector is exactly the same as the single SOF detector. The paired probe consists of a plastic scintillator with and without LiF or boron as a neutron converter

All measurement data presented in this paper were obtained using the paired and single SOF detector systems. The components of the paired system are similar to the former SOF detector system described in our earlier work [9]. Fig. 1 shows a schematic diagram of the paired SOF detector system. The other components of the SOF detector include optical fibers, photon counting units and counters. The photon-counting unit (Hamamatsu H7155) consists of a photo-multiplier tube, a pre-amplifier and a discriminator. In this research, we used a BC490 plastic scintillator manufactured by Bicron Ltd. The BC490 is partially polymerized and hardened with a catalyst which makes it possible to be tightly connected to the tip of a plastic optical fiber (Mitsubishi Rayon MH4002, 1 mm-diameter optical fiber with 2.2 mm-diameter polyethylene shielding). A small amount of LiF powder (enriched 95 % ^6^Li) was mixed with one of the plastic scintillators. The reactions between ^6^Li nuclei and thermal neutrons emit charged particles (alpha and triton), which produce scintillation photons in the plastic scintillator.

The photon signals are relayed through the optical fiber to the Photon Counting Unit and then converted into 30 ns-width TTL pulses. The pulse counts are sent to a personal computer via a universal serial bus (USB) connection. When using a plastic scintillator for counting neutrons, the signals in the detector can be produced by alpha particles, Li nuclei, recoil protons and gamma rays. It is necessary to clearly differentiate the signals produced by these particles in order to correctly estimate thermal neutron flux. The signal from gamma rays can be the main source of noise for SOF detector. Although a plastic scintillator hardly causes photoelectric effect, a signal from the Compton scatter of high-energy gamma rays contributes significantly to the total signal measured by the detector. This gamma ray contribution can be minimized if a very small detector is used for measurement. To account for the gamma ray and fast neutron signals, scintillators without ^6^LiF were used, which include only gamma ray and fast neutron signals. The contributions from the gamma rays and fast neutrons can be delineated and corrected based on the difference between the signals obtained from the scintillator without ^6^LiF and the signals from the scintillator with ^6^LiF.

In a few cases, a single SOF detector has been adopted to increase the number of monitoring points. A single SOF detector has almost the same composition and characteristics as the paired detector except for the gamma-ray and fast-neutron signal correction. Since the thermal neutron flux measured by the single SOF detector was similar as that of the paired detector, the single SOF was as effective as a relative neutron fluence monitor. Furthermore, because the cross-section of ^6^Li is almost proportional to that of ^10^B for neutron energy range in reactor-based BNCT, the reaction rate of ^6^Li is proportional to the ^10^B dose in the tumor.

### SOF measurement in clinical use

**Fig. 2:**
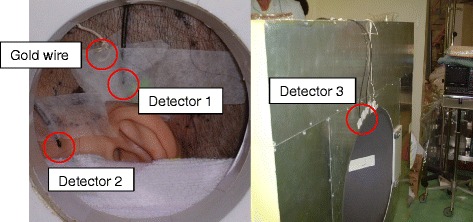
SOF detectors arrangement in clinical use during BNCT. The SOF detectors are placed at the center of ROI (detector 1), peripheral of interest (detector 2) and in front of the collimator (detector 3) without disturbing the gold wires

Clinical trial measurements with the SOF detector started in December 2002. By the end of 2006, we had conducted a total of 16 clinical measurements. In clinical measurements, the SOF detectors were placed at the center of the region of interest (ROI) (detector 1), peripheral of interest (detector 2) and in front of collimator (detector 3) as shown in Fig. 2. These detectors were used as a thermal neutron flux monitor at the ROI (detector 1) and as a patient’s motion monitor (detector 2) and reactor power fluctuation monitor (detector 3). In all BNCT treatments, the gold wire activation method was the primary method for estimating thermal neutron flux. The SOF detectors were placed without disturbing the gold wire measurement. Thermal neutron irradiation was used for treating skin melanoma. For patients with glioblastoma, parotid cancer and fibrosarcoma, epithermal neutrons beams were used. Here, a paired SOF probe was mainly used for neutron fluence monitoring while a single SOF probe was used for patient-motion detection.

### Calibration

In the present work, efficiency for each detector varied due to non-identical sizes of the scintillator, transmission loss of optical fiber, gain of photo-multiplier tubes and slightly different discrimination level. Correction factors for the measured counts by each detector were established in order to account for the difference in the relative efficiencies of the detectors. The measured counts for the scintillator with and without ^6^LiF were assumed to be expressible in terms of Eqs. (1) and (2), respectively as

1 $$ {C}_{+}={R}_{n+}\cdot {F}_n+{R}_{g+}\cdot {F}_g+{R}_{f+}\cdot {F}_f $$

2 $$ {C}_{-}={R}_{g-}\cdot {F}_g+{R}_{f-}\cdot {F}_f $$

In Eq. (1), C_+_ represents the measured counts of the scintillator with ^6^LiF. *F*_*n*_, *F*_*g*_, and *F*_*f*_ are the particle fluence for neutrons, gamma rays and fast neutrons respectively. *R*_*n+*_, *R*_*g+*_, and *R*_*f+*_ are the detector response factor for thermal neutrons, gamma rays and fast neutrons respectively. In Eq. (2), *C*_*−*_*, R*_*g-*_*,* and *R*_*f-*_ are the measured counts, response factor for gamma rays and fast neutrons, respectively, for the scintillator without ^6^LiF.

3 $$ {C}_{+}^{\hbox{'}}={R}_{g+}\cdot {F}_g^{\hbox{'}} $$

4 $$ {C}_{-}^{\hbox{'}}={R}_{g-}\cdot {F}_g^{\hbox{'}}. $$

Here, *C’*_*+*_ and *C’*_*−*_ are the measured gamma ray counts for the detectors with and without the neutron converter, respectively. The response factors *R*_*g+*_ and *R*_*g-*_ were obtained from the measured counts of both detectors when only a gamma-ray field was used following Eqs. (3) and (4). These correction factors were determined from measurements using an intense pure gamma-ray source such as ^137^Cs.

The response ratio *R*_*g+*_*/R*_*g-*_ can be expressed in terms of *C’*_*+*_ and *C’*_*−*_ from Eqs. (3) and (4). Since the detector response depends only on the scintillator volume, the response factors *R*_*f+*_ and *R*_*f-*_ should be proportional to *R*_*g+*_ and *R*_*g-*_*,* respectively. We obtain *C*_*+*_ in the form of Eq. (5).

5 $$ {C}_{+}={R}_n\cdot {F}_n+\frac{R_{g+}}{R_{g-}}\cdot {C}_{-}={R}_n\cdot {F}_n+\frac{C_{+}^{\hbox{'}}}{C_{-}^{\hbox{'}}}\cdot {C}_{-} $$

The expression for the neutron flux *F*_*n*_ can then be deduced from Eq. (5) and is given by Eq. (6).

6 $$ {F}_n=\frac{C_{+}-\frac{C_{+}^{\hbox{'}}}{C_{-}^{\hbox{'}}}\cdot {C}_{-}}{R_{n+}} $$

Similarly, we can also calculate the uncertainties for the thermal neutron flux, using the relative standard deviation given by,

7 $$ \frac{\sigma_{F_n}}{F_n}=\frac{\frac{1}{\sqrt{C_{+}}}\sqrt{1+{\left(\frac{R_{g+}}{R_{g-}}\right)}^2\frac{C_{-}}{C_{+}}}}{1-\frac{R_{g+}}{R_{g-}}\frac{C_{-}}{C_{+}}} $$

### Expected fluence and estimation error

When the neutron fluence at any arbitrary time of irradiation is known, we can calculate the neutron fluence at the required time by using simple rules of mathematics. In our case, the first 15 min value of the neutron fluence was obtained and referred as the “pull-out” value *F*_*SOF, pull-out*_. On the basis of the pull-out value, we can calculate the neutron fluence during the total irradiation time *T*_*irrad*_ and referred as the Expected fluence *F*_*SOF,exp*_ given by

8 $$ {F}_{SOF,\; exp}=\frac{T_{irrad}}{T_{pull- out}}\cdot {F}_{SOF, pull- out} $$

The neutron fluence obtained from Eq. (8) is the calculated neutron fluence during the entire irradiation time. The expected neutron fluence is a calculated mathematical value which may differ from that of the observed neutron fluence. During clinical trials, the neutron fluence measured by the SOF detector may be slightly different from that the expected value. This difference in the expected fluence *F*_*SOF,exp*,_ and the observed fluence *F*_*SOF, Final*_, gives rise to an error referred as the estimation error *E*_*SO*F_. The error was obtained using the percent error formula given below.

9 $$ {E}_{SOF}=\frac{F_{SOF,\kern0.5em  Final}-{F}_{SOF,\; exp}}{F_{SOF,\  exp}} $$

Similarly, the estimation error for the gold wire *E*_*AU*_ is given by

10 $$ {E}_{Au} = \frac{F_{Au,\kern0.5em  Final} - {F}_{Au,\  exp}}{F_{Au,\  exp}} $$

Here, the full irradiation time was determined from constraints of skin dose or vascular dose and minimum tumor dose. The “pull-out” and “final” values were based on the SOF detector placed on the patient skin.

## Results

### Clinical measurements

Table 1 shows a summary of the BNCT clinical measurements where the SOF detector was used. It is evident from Table 1 that the neutron fluence estimated by the gold wire activation method differs from that obtained with the SOF detectors. The neutron fluence obtained by the SOF detector was in better agreement with the expected fluence than the gold wire activation. Thus, the estimation errors for the SOF detector were found to be smaller in most cases compared to the gold wire method. The estimation error for the SOF detector and gold wire was calculated using (9) and (10), respectively. The highest estimation error for the SOF detector was observed in cases 4 and 14 and the lowest occurred in cases 6 and 13. The estimation errors were based on the neutron flux monitor at the ROI. In case 10, irradiation was performed during surgery (craniotomy) and the SOF detector was placed on the edge of the collimator instead of the patient skin. The estimation error in this case was based on the neutron flux monitor at the ROI. Similarly, in case 12, the SOF detector was put under the patient’s right ear tube. Thus, in cases 10 and 12, the estimation error is not based on the neutron flux monitor at the ROI.

**Table 1:** A summary of clinical trials in BNCT

No.	Tumortype	Reactor (Mode)	Irrad. time [min.]	Probe type	Fluence [n/cm^2^]				Expected fluence [n/cm^2^]	Estimation error (%)	
					SOF detector		Gold wire			Gold	SOF
					Pull-out	Final	Pull-out	Final			
1	G	KUR(E)	120	BP	1.01 × 10^11^	7.72 × 10^11^	1.12 × 10^11^	9.58 × 10^11^	8.93 × 10^11^	7.26	−4.46
2	G	KUR(E)	60	BP	1.29 × 10^11^	5.02 × 10^11^	1.17 × 10^11^	4.90 × 10^11^	4.68 × 10^11^	4.62	−2.68
3	F	KUR(E)	80	LS	2.18 × 10^11^	1.17 × 10^12^	2.09 × 10^11^	1.20 × 10^12^	1.11 × 10^12^	7.76	0.55
4	P	KUR(E)	83	LS	1.39 × 10^11^	6.22 × 10^11^	1.14 × 10^11^	4.82 × 10^11^	6.30 × 10^11^	−23.46	−18.66
5	G	KUR(E)	90	LS	1.18 × 10^11^	6.92 × 10^11^	2.03 × 10^11^	1.09 × 10^12^	1.22 × 10^12^	−10.22	−2.18
6	G	KUR(E)	87	LS	1.24 × 10^11^	7.25 × 10^11^	1.36 × 10^11^	7.86 × 10^11^	1.63 × 10^12^	−0.35	0.47
7	G	KUR(E)	90	LS	1.41 × 10^11^	8.27 × 10^11^	3.01 × 10^11^	1.93 × 10^12^	1.80 × 10^12^	6.89	−2.27
8	G	KUR(T)	60	LP	1.55 × 10^11^	6.02 × 10^11^	3.17 × 10^11^	1.21 × 10^12^	1.27 × 10^12^	−4.26	−2.82
9	M	KUR(T)	90	LP	7.86 × 10^11^	4.60 × 10^12^	9.27 × 10^10^	5.89 × 10^11^	5.56 × 10^11^	5.83	−2.46
10^a^	G	JRR4(E)	69	LP	2.64 × 10^11^	1.03 × 10^12^	3.05 × 10^12^	1.25 × 10^13^	1.41 × 10^13^	−11.10	−4.86
11	G	JRR4(E)	34	LP	4.19 × 10^11^	8.62 × 10^11^	3.14 × 10^12^	7.03 × 10^12^	7.13 × 10^12^	−1.43	−0.67
12^b^	G	JRR4(E)	32	LP	1.31 × 10^10^	2.68 × 10^10^	3.05 × 10^12^	6.49 × 10^12^	6.52 × 10^12^	−0.38	−3.30
13^c^	M	JRR4(T)	75	LP	6.61 × 10^11^	3.00 × 10^12^	8.30 × 10^11^	4.59 × 10^12^	4.26 × 10^12^	7.83	−0.15
14	G	KUR(E)	90	LP	2.08 × 10^11^	1.17 × 10^12^	3.11 × 10^11^	1.91 × 10^12^	1.87 × 10^12^	2.02	−6.28
15^d^	P	JRR4(E)	25	LL	4.43 × 10^11^	1.06 × 10^12^	2.91 × 10^12^	4.77 × 10^12^	4.85 × 10^12^	−1.61	0.65
16^d^	G	JRR4(E)	29	LL	5.76 × 10^11^	1.54 × 10^12^	2.93 × 10^12^	5.51 × 10^12^	5.67 × 10^12^	−2.84	−1.82

In Tumor Type column, G,P,M and F stand for Gliobrastoma, Parotid cancer, Melanoma and Fibro sarcoma, respectively

In Reactor (Mode) column, (E) and (T) stand for epi-thermal neutron and thermal neutron irradiation respectively

In the Probe Type column, BP, LS, LP and LL stand for paired boron-loaded, single lithium mixed, paired lithium mixed and loop-type lithium mixed, respectively

^a^Irradiation performed during craniotomy, SOF detector placed on the edge of the collimator instead of patient skin

^b^SOF detector placed under patient ear tube

^c^Gold wire placed on the patient skin (Except case 14 all cases in JRR, gold wire placed inside the port)

^d^First 10 min used for calculating the expected fluence

### A real-time monitoring by SOF detector in BNCT treatment

**Fig. 3:**
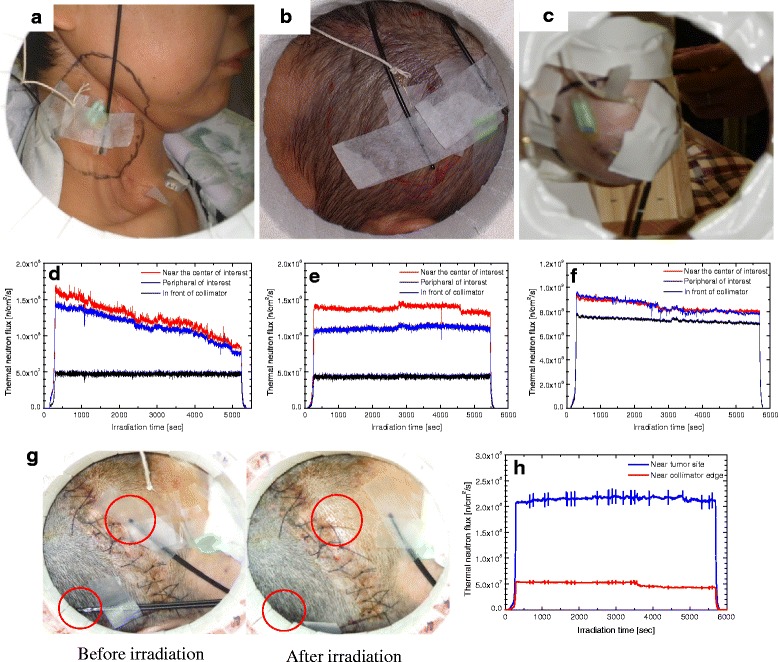
The monitoring position of the SOF detectors during BNCT at case (**a**) 4 (**b**) 6 (**c**) 9 (**g**) 14 (SOF probe before and after irradiation). **d**, **e**, **f** and **h** show the real-time measurements of the thermal neutron flux by the SOF detector during BNCT for cases 4, 6, 9 and 14, respectively

The monitoring position of the SOF detector for cases 4, 6, 9 and 14 are shown in Fig. 3a,b,c and g, respectively. Similarly, Fig. 3d,e,f and h show the SOF detector working effectively as a real-time thermal neutron monitor in clinical BNCT for cases 4,6,9 and 14, respectively. From the real-time monitor we noted that any changes in the neutron flux monitor of detector 2 and 3 affected the neutron flux measured by detector 1.

According to Table 1, in case 4, it was realized that the total amount of irradiation was 18.66 % lower than expected. The decline in the thermal neutron flux was observed in real-time as shown in Fig. 3d. Both the output of the monitor at the center of the ROI and the monitor near the edge of the ROI declined continuously. However, the same drastic change in the thermal flux was not observed by another SOF monitor placed in front of the collimator. This was the highest decline in the neutron flux in the entire clinical measurements. The continuous decline in the neutron flux shown by detector 1 was most probably due to the change in the position of the patient during irradiation.

Errors seem to decrease in cases 6 and 9. In case 6 (Fig. 3e), the neutron flux monitor in front of the collimator remained fairly constant. The estimation error 0.47 % in this case was most probably due to the slight change in the flux at the peripheral of the ROI. On the other hand, in case 9 (Fig. 3f) both the monitor in front of the collimator and near the periphery of interest showed decline in the thermal neutron flux. Therefore, in this case the neutron flux at the ROI was affected by the patient motion and reactor power fluctuation. In all above cases, the dislocation of SOF detector from the original position was not observed.

Figure 3g shows the location of the SOF detector probe before and after irradiation for case 14. As shown in Fig. 3g, the probe was inside a red circle before irradiation; however, after irradiation the probe was located outside of the red circle. The decline in neutron flux near the edge of the collimator was observed after 3,600 s of irradiation. Similarly, the real-time monitor at ROI showed decline in the flux after 4,800 s of irradiation. In this case, the dislocation of the probe from the original position affected the SOF measurements.

### Measurement uncertainty of gold wire and SOF detector

The measurement uncertainty for the SOF detector can be deduced from Eqs. (1), (2), (6) and (7). From Eq. (7), the uncertainties for thermal neutron flux at 10^8^ n/cm^2^/s and 10^9^ n/cm^2^/s are 0.53 % and 0.17 %, respectively. Here, we used parameters for response ratio R_g+_/R_g-_ = 1, gamma-ray contribution ratio C_−_/C_+_ = 0.1 and the response factor R_n+_ = 2,072 n/cm^2^/counts from our previous work [6]. The estimated uncertainty does not contain calibration uncertainty for absolute measurement. Similarly, measurement uncertainties for the gold wire activation method at KURRI and JRR4 were estimated as 5.82 % ± 1.73 % and 1.32 % ± 0.40 %, respectively for estimating thermal neutron flux at the beginning. The gold wire measurements at KURRI were performed on the patient skin where the average thermal neutron flux was 3.21 × 10^8^ n/cm^2^/s. At JRR4, gold wire measurements were performed inside the beam port where the average thermal neutron flux was 3.83 × 10^9^ n/cm^2^/s. This is 12 times higher than at KURRI. The measurement acquisition times for the activation were 60 s at KURRI and 103.8 s (30–200 s) at JRR4.

## Discussion

From the result section, we observed that the real-time monitoring of the thermal neutron distribution was possible with the help of the SOF detector. The gold wire method cannot be used for the same purpose because it provides only retrospective and integrated information on the neutron flux distribution. The difference in the estimated neutron fluence by the gold wire method and SOF detector was likely due to the difference in the location of the detector and the gold wire. The gold wire was placed on the patient skin at KURRI and inside the beam port at JRR4. Even at the skin surface, the position of the SOF detector did not align exactly with the position of gold wire 1 (pulled out after 15 min) or gold wire 2 (irradiated to the end of the treatment).

Generally, the gold wire is pulled out after the first 15 min of irradiation. The location of the SOF detector and the gold wire is also different (in case of JRR 4, gold wire was placed inside the port) and even on the patient skin the position of the SOF detector did not align exactly with the position of gold wire 1. This makes direct comparison between the SOF detector and gold wire measurement difficult. Similarly, TLD is only useful for gamma dosimetry. Thus, in the current research the neutron flux measured by the SOF detector was compared with the expected fluence because it was similar to that of the treatment planning system. However, the test level of the SOF detector can be further improved by comparing the results with simulation techniques which are capable of modeling complex geometries.

### Issues and solutions in the clinical use of the scintillator with optical fiber (SOF) detector

We observed that the measurement uncertainty of the SOF detector was quite small and its contribution in SOF detector estimation error was not of much significance. The main source of error in SOF detector measurement at the ROI is due to the variation in the reactor condition, patient motion and dislocation of the SOF detector from the original position. Thus, the measured data should be carefully reviewed.

**Fig. 4:**
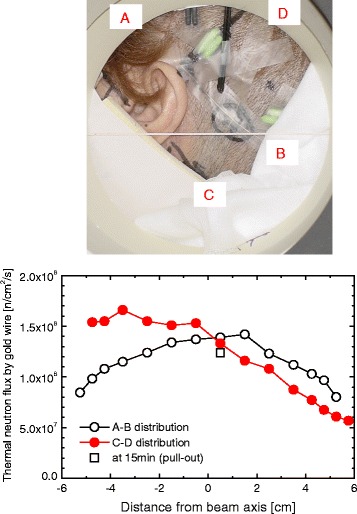
Thermal neutron flux distribution measured by gold wire for case 1. The gradient of thermal neutron flux differs according to the measured position

It was shown that change in the positioning of the patient affected the SOF measurements. Thus, for calculating accurate tumor dose the patient positioning should always be monitored continuously. Figure 4, shows the distribution of thermal neutron flux for case 1 measured by the gold wire. Here, the gradient of thermal neutron flux differs according to the measured position. Generally, the gradient of thermal neutron flux near the collimator edge is relatively larger than at the center. Therefore, the placement of the SOF detector near the collimator edge is more effective for monitoring patient movement during irradiation. Even though the patient has moved during irradiation, skin dose at the SOF detector position can be assessed in real-time. Similarly, the fluctuation of the reactor power directly affects the tumor dose and skin dose. The fluctuation of the reactor power can be monitored stably at the site where measurement is not affected by patient motion such as in front of the collimator or inside the beam port.

The displacement of the SOF detector probe from the patient skin happened during treatment in case 14 as shown in Fig. 3g. Due to the displacement of the probe, it was impossible to estimate the measured position and thus the measured value will be of less importance. A strong adhesive tape could not be used during treatment since it was harsh on the patient’s skin. The displacement of the probe also occurred as the adhesive tape was weakened by patient’s sweat during treatment. Close adhesion to the skin is especially difficult for a patient who had a craniotomy because of difficulty of shaving around the irradiated area. The neutron flux decreases as the distance from the central beam axis increases, as shown in Fig. 4. Thus, in case 14, the SOF detector displaced away from the beam axis (Fig. 3g) during irradiation and a small decline in the neutron flux was recorded by the real-time monitor (Fig. 3h) of detector 1 and 2 around 4,800 and 3,600 s, respectively. This affected the accuracy of the detected neutron flux and consequently affected the SOF measurement.

**Fig. 5:**
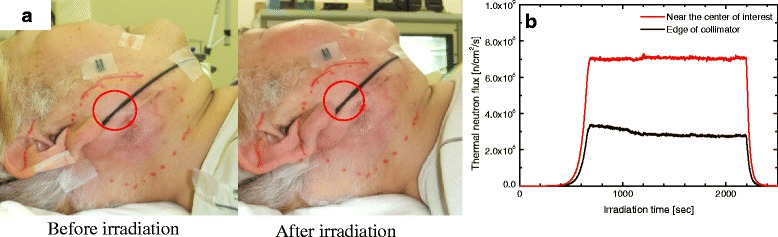
**a** Loop type detector (before and after irradiation) for case 15. It remained inside the red circle during the entire irradiation. **b** The real-time monitor of the loop type SOF detector during BNCT treatment

To overcome the displacement issue, we developed a loop type probe as shown in the Fig. 5. Originally, the SOF probe consisted of two identical optical fibers with scintillators. The two scintillators were constructed such that they face each other. Since the loop-type probe can be fixed from two directions, it is expected that a measurement error due to the probe detachment during treatment would be minimized. The loop type probe was adapted in cases 15 and 16. Figure 5, shows that the SOF probe remained in its original position during the entire irradiation and also the significant change in the neutron flux was not observed in the real-time monitor.

### SOF detector at low neutron field

**Fig. 6:**
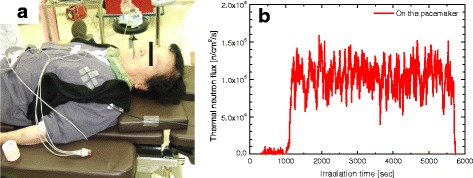
The monitoring position and a real-time measurement by the SOF detector on the pacemaker for case 13. **a** Position of SOF detector on the pacemaker. **b** Real- time measurement by the SOF detector on the pacemaker. The SOF detector is capable of measuring the thermal neutron flux even of the order 10^5^ n/cm^2^/s

The monitoring position of the SOF detector at the pacemaker is shown in Fig. 6a. A 5-mm-thick thermal neutron shield sheet containing B_4_C was used to cover the pacemaker. This helps to reduce the thermal neutron flux in the pacemaker region. The thermal neutron flux measured at the pacemaker position was fluctuating between 10^4^ and 10^5^ n/cm^2^/s, as shown in Fig. 6b, where the graph was plotted as a 30-seconds moving average. The fluctuation in the measurement was larger compared to the flux at 10^8^ n/cm^2^/s. The total thermal neutron fluence on the pacemaker position was obtained as 4.74 × 10^8^ n/cm^2^. This confirms that the SOF detector is able to measure thermal neutron flux as low as 10^5^n/cm^2^/s. The gold wire may take a few days to measure the same order of thermal neutron flux.

Soft error rate (SER) may be induced when a semiconductor device on a pacemaker is subjected to irradiation.. The maximum tolerable cumulative radiation dose for safe operation of a pacemaker depends highly on the pacemaker type, model and the dose rate [14]. SER of a semiconductor device due to thermal neutron flux of 10^5^ n/cm^2^/s from a nuclear reactor is about 5 times higher than environmental level thermal neutron flux [15]. Since, the SER of the environmental thermal neutron flux is very low, the potential effect on the pacemaker by the thermal neutron field may have been negligible in this case. However, the current manuscript does not aim to determine the safe irradiation limit of pacemakers during BNCT treatment.

### Comparison between boron loaded and LiF mixed SOF detector

**Fig. 7:**
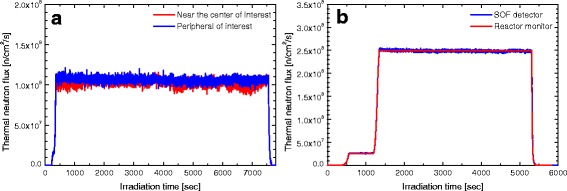
The results of real-time monitoring by (**a**) a paired boron-loaded SOF detector for case 1 and (**b**) a paired LiF-mixed SOF detector for case 10. Fluctuation in thermal neutron flux for LiF-mixed probe was less in comparison with the boron-loaded probe and the SOF measurements were in good agreement with the reactor monitor

Figure 7a,b show the measurement results for the paired boron-loaded probe (Case 1) and the paired LiF-mixed probe (Case 10), respectively. The fluctuations in the thermal neutron flux measured by the boron-loaded and the LiF-mixed probes were 2.85 % at 1.1 × 10^8^ n/cm^2^/s (2.94 × 10^4^ cps) and 0.71 % at 2.5 × 10^8^ n/cm^2^/s (1.12 × 10^5^ cps), respectively. If the LiF-mixed probe was used at 1.1 × 10^8^ n/cm^2^/s, the expected fluctuation will be 1.07 %. This indicates that the measurement using LiF-mixed probe was more stable than the boron-loaded probe. In case 10, the estimation error based on reactor monitor was −1.59 % indicating that the SOF measurement was in good agreement with the reactor monitor. Cases 1 and 10 showed that the LiF-mixed probe measurements were less fluctuating than the boron-loaded probe. This was due to the fact that when LiF (enriched 95 % ^6^Li) interacts with a neutron it emits a large Q-value and two energetic particles: a triton particle (2.05 MeV) and an alpha particle (2.73 MeV). The light yielded from triton is nearly a factor of 10 times higher than that of the 1.5 MeV alpha particle from neutron capture on ^10^B[16]. Thus, we used the boron-loaded probe only in the first 2 cases and in the remaining 14 cases the LiF mixed probe was used.

## Conclusion

Clinical measurements using the SOF detector to measure thermal neutron flux during BNCT treatment confirmed that SOF detectors were effective as a real-time thermal neutron monitor. The real-time monitoring of the thermal neutron by SOF detector suggests that the neutron flux distribution and intensity at the ROI vary due to the reactor condition, patient motion and dislocation of the SOF detector. To minimize the detector displacement problem, a loop-type probe was developed. The authors believe the presented work will contribute towards the field of on-line neutron monitoring in clinical BNCT irradiation.

## Abbreviations

^10^B, boron; ^7^Li, lithium; BNCT, boron neutron capture therapy; JRR4, Japan Atomic Energy Agency reactor No.4; KUR, Kyoto University Reactor; KURRI, Kyoto University Research Reactor Institute; LET, linear energy transfer; LiF, lithium fluoride; ROI, region of interest; SER, soft error rate; SOF, scintillator with optical fiber; TTL, transistor-transistor logic; USB, universal Serial Bus; α, alpha
